# Intact fibroblast growth factor 23 levels and outcome prediction in patients with acute heart failure

**DOI:** 10.1038/s41598-021-94780-7

**Published:** 2021-07-30

**Authors:** Anne Cornelissen, Roberta Florescu, Kinan Kneizeh, Christian Cornelissen, Vincent Brandenburg, Elisa Liehn, Alexander Schuh

**Affiliations:** 1 Department of Cardiology, Angiology and Internal Intensive Medicine, University Hospital Aachen, RWTH Aachen University, Aachen, Germany; 2 Department of Pneumology, University Hospital Aachen, RWTH Aachen University, Aachen, Germany; 3 Department of Cardiology and Nephrology, Rhein-Maas Klinikum, Wuerselen, Germany; 4 Department of Internal Medicine I, St. Katharinen Hospital Frechen, Kapellenstrasse 1-5, 50226 Frechen, Germany

**Keywords:** Biomarkers, Cardiology, Risk factors

## Abstract

Elevated fibroblast growth factor 23 (FGF23) levels are associated with adverse outcome in populations with cardiovascular disease and chronic kidney failure. It is unclear if FGF23 has significance in prognosis estimation in patients with acute heart failure (HF) when compared to traditional risk estimation tools. Serum levels of intact FGF23 were assessed in 139 patients admitted to the Intermediate Care Unit of a tertiary hospital for acute HF. Patients were followed-up for one year. After exclusion of patients who were lost to follow-up, data outliers, and patients with sampling errors, the final study cohort comprised 133 patients. The Seattle Heart Failure (SHF) Model was used to estimate one-year survival. FGF23 levels correlated with HF severity and were strongly associated with one-year mortality. Associations between one-year outcome and FGF23, assessed on day 1 after admission, were still evident after multivariable adjustment (OR 15.07; 95%CI 1.75–129.79; *p* = 0.014). FGF23 levels predicted the one-year outcome with similar accuracy as the SHF Model, both if assessed on day 1 and on day 2 after admission (FGF23d1: AUC 0.784; 95%CI 0.669–0.899; FGF23d2: AUC 0.766; 95%CI 0.631–0.901; SHF: AUC 0.771; 95%CI 0.651–0.891). The assessment of FGF23 in patients with acute HF might help identify high-risk patients that are more prone to complications, need a closer follow-up and more aggressive treatment.

## Introduction

Despite major advances in diagnostics and therapy options, cardiovascular diseases (CVD) are still associated with a high morbidity and mortality worldwide, especially if the onset is acute. Multiple tools for risk estimation are available and usually consist of a set of clinical parameters, often combined with imaging results, summarized in a risk score. In acute situations, however, the assessment of complex risk scores is often time-consuming and therefore not feasible in daily routine. The identification of singular biomarkers that are capable of accurately predicting an individual’s risk might help identify patients who need a more aggressive treatment strategy and a closer follow-up.

Fibroblast growth factor 23 (FGF23) is a phosphaturic hormone mainly produced in the bone that controls mineral homeostasis by regulating serum phosphate, parathyroid hormone, and 1,25-(OH)_2_-vitamin D_3_. FGF23 has been identified as an early biomarker of impaired kidney function, as FGF23 levels rise prior to the development of hyperphosphatemia, 1,25-(OH)_2_D_3_ deficiency, hypocalcemia, or secondary hyperparathyroidism^1,2^. Chronic kidney disease (CKD) is associated with an increased risk of CVD, and cardiovascular event rate increases as eGFR declines^3^. FGF23 has been proposed as sensitive biomarker for adverse renal and extrarenal outcomes^4,5^. Heart failure (HF) is the second most common cause of secondary FGF23 excess. FGF23 rises during HF exacerbations, associates with disease severity, and predicts risk of HF-related death and hospitalization, as well as likelihood of therapeutic response to angiotensin-converting enzyme inhibitor (ACE inhibitor) therapy irrespective of kidney function^6–9^. However, while recent data indicate that the prognostic impact of FGF23 in chronic HF is limited^10^, less is known about the prognostic value of FGF23 levels obtained shortly after hospitalization for acute HF in critically ill patients.

FGF23 has been shown to be produced and released by cardiac fibroblasts in response to acute myocardial damage^11^. Considering that FGF23 can be rapidly obtained from a single blood draw, we hypothesized that this biomarker might be valuable for prognosis estimation especially in patients with acute HF where timely decision-making is mandatory. In this single-center study, we assessed serum levels of intact FGF23 in 139 patients who had been admitted to the Intermediate Care Unit of University Hospital Aachen for acute HF. We sought to correlate FGF23 to clinical and echocardiographic parameters indicating HF severity. Furthermore, we aimed to assess the capability of FGF23 to predict the one-year mortality in comparison with the well-established Seattle Heart Failure (SHF) Model.

## Results

### Study population and patient characteristics

A total of 139 patients admitted to the Intermediate Care Unit of University Hospital Aachen for acute HF met the initial inclusion criteria of the study. After exclusion of patients lost to follow-up, patients with incomplete assessments of FGF23 levels, and outliers with extremely high FGF23 levels, the final study cohort encompassed a total of 133 patients (Supplementary Fig. S1).

Patient characteristics are shown in Supplementary Table S1. Mean age was 66.98 ± 12.22 years (range 32–94), and 96 patients (72.2%) were male. From all patients with acute HF on admission, 37 patients (27.8%) had previously known HF (chronic decompensated HF), whereas de novo HF was diagnosed in 96 patients (72.2%). 9 patients (6.8%) were diagnosed with HFpEF, 58 patients (43.6%) were classified as HFmrEF, and 66 patients (49.6%) had HFrEF. 50 patients had no limitations of physical activity before admission (NYHA I, 37.6%), 38 patients had slight limitations (NYHA II, 27.8%), 29 patients reported a marked limitation of physical activity (NYHA III, 21.8%), and 17 patients were unable to perform any physical activity without discomfort (NYHA IV, 12.8%).

110 patients (82.7%) survived, and 23 patients (17.3%) died within one year after admission.

### Associations between FGF23 levels and LVEF

**Figure 1:**
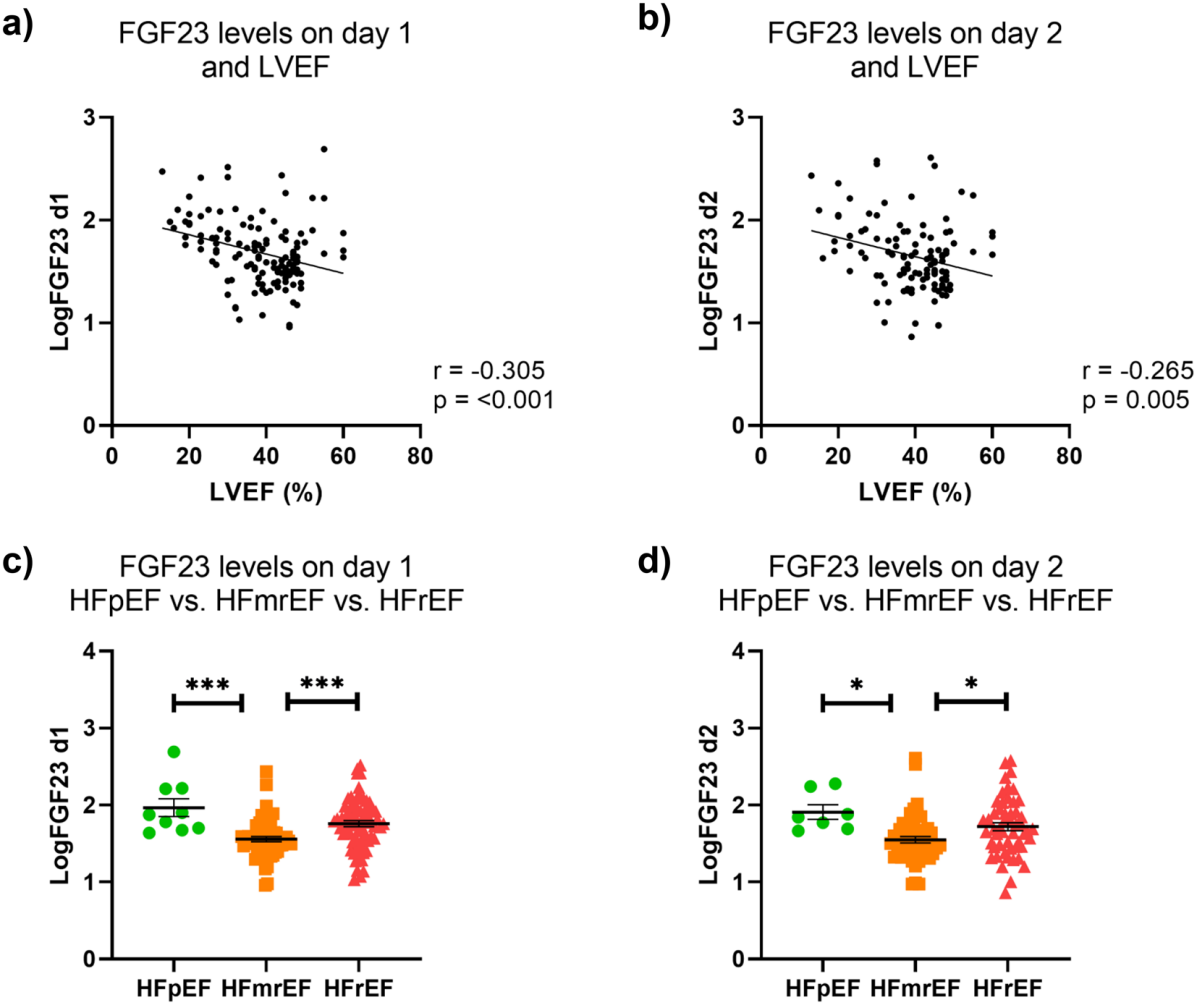
Relationship between LVEF and FGF23 levels. (**a**, **b**) In an unadjusted analysis, FGF23 levels, assessed on day 1 (**a**) and on day 2 (**b**) showed a modest inverse correlation with LVEF in patients with acute HF. (**c**, **d**) Patients who were diagnosed with HFpEF or HFrEF had significantly higher FGF23 levels compared with HFmrEF patients, both when FGF23 was assessed on day 1 (**c**) and on day 2 (**d**).

Serum levels of intact FGF23 were assessed at admission (day 1) and on the following day (day 2). There was a modest inverse correlation between logFGF23 levels and LVEF, both on day 1 (r = − 0.31, *p* =  < 0.001) and on day 2 (r = − 0.27, *p* = 0.005) (Fig. 1a,b). However, after controlling for age, NT-proBNP, and eGFR, the correlation between logFGF23 levels and LVEF lost significance (day 1: r = − 0.09; *p* = 0.338; day 2: r = 0.007; *p* = 0.942, Table 1). Table 2 shows mean logFGF23 levels in patients with HFpEF, HFmrEF, and HFrEF with and without adjustment for age, NT-proBNP, and eGFR in a one-way between-groups analysis of covariance. Patients with HFpEF and HFrEF had significantly higher logFGF23 levels compared to patients with HFmrEF, both if assessed on day 1 (Fig. 1c) and on day 2 (Fig. 1d). After adjustment for age, NT-proBNP, and eGFR, logFGF23 levels on day 1 were still significantly higher in patients with HFpEF compared to patients with HFmrEF, while significance was lost for the comparison between HFmrEF and HFrEF. There were no significant differences in logFGF23 levels on day 2 among HFpEF, HFmrEF, and HFrEF patients when age, NT-proBNP, and eGFR were controlled for.

**Table 1:** Correlation between FGF23 levels and parameters indicating HF severity.

	LogFGF23 day 1		LogFGF23 day 1*		LogFGF23 day 2		LogFGF23 day 2*	
	Correlation	*p *value	Correlation	*p* value	Correlation	*p* value	Correlation	*p* value
**Echocardiography**								
LVEF	− 0.305	< 0.001	− 0.087†	0.338†	− 0.265	0.005	0.007†	0.942†
LVEDD	0.423	< 0.001	0.251	0.014	0.352	0.001	0.139	0.219
LVESD	0.447	< 0.001	0.282	0.006	0.377	< 0.001	0.188	0.099
Septum thickness	− 0.024	0.806	0.029	0.781	− 0.087	0.414	− 0.019	0.863
LA Area	0.444	< 0.001	0.292	0.004	0.367	< 0.001	0.203	0.072
DT	− 0.230	0.043	− 0.095	0.438	− 0.205	0.091	0.028	0.832
E/A	0.225	0.052	0.171	0.171	0.256	0.038	0.119	0.372
E/E’	0.407	< 0.001	0.228	0.062	0.502	< 0.001	0.339	0.008
RVSP	0.327	0.005	0.170	0.177	0.248	0.047	0.069	0.607
TAPSE	0.040	0.689	0.111	0.291	0.031	0.778	0.073	0.528
**Laboratory/clinical parameters of HF severity**								
NT-pro-BNP	0.485	< 0.001	0.252‡	0.005‡	0.588	< 0.001	0.416‡	< 0.001‡
NYHA	0.410	< 0.001	0.214	0.018	0.409	< 0.001	0.216	0.030
Killip	0.257	0.003	0.110	0.226	0.181	0.057	− 0.016	0.877

*Adjusted for age, NT-proBNP, eGFR, and LVEF.

^†^Adjusted for age, NT-proBNP, and eGFR.

^‡^Adjusted for age, eGFR, and LVEF.

**Table 2:** LogFGF23 in HFpEF, HFmrEF, and HFrEF.

logFGF23 levels	HFpEF (mean ± SD)	HFmrEF (mean ± SD)	HFrEF (mean ± SD)	*p* value HFpEF vs. HFmrEF	*p* value HFpEF vs. HFrEF	*p* value HFmrEF vs. HFrEF
Day 1	1.97 ± 0.35	1.58 ± 0.26	1.76 ± 0.32	0.0005	0.1193	0.0008
Day 1*	1.88 ± 0.08	1.65 ± 0.04	1.71 ± 0.03	0.0360	0.1490	0.6990
Day 2	1.91 ± 0.25	1.57 ± 0.33	1.72 ± 0.37	0.0248	0.3519	0.0285
Day 2*	1.78 ± 0.10	1.67 ± 0.04	1.65 ± 0.04	0.9999	0.6350	0.9999

*Adjusted for age, NT-proBNP, and eGFR.

### FGF23 levels are associated with clinical severity of heart failure

**Figure 2:**
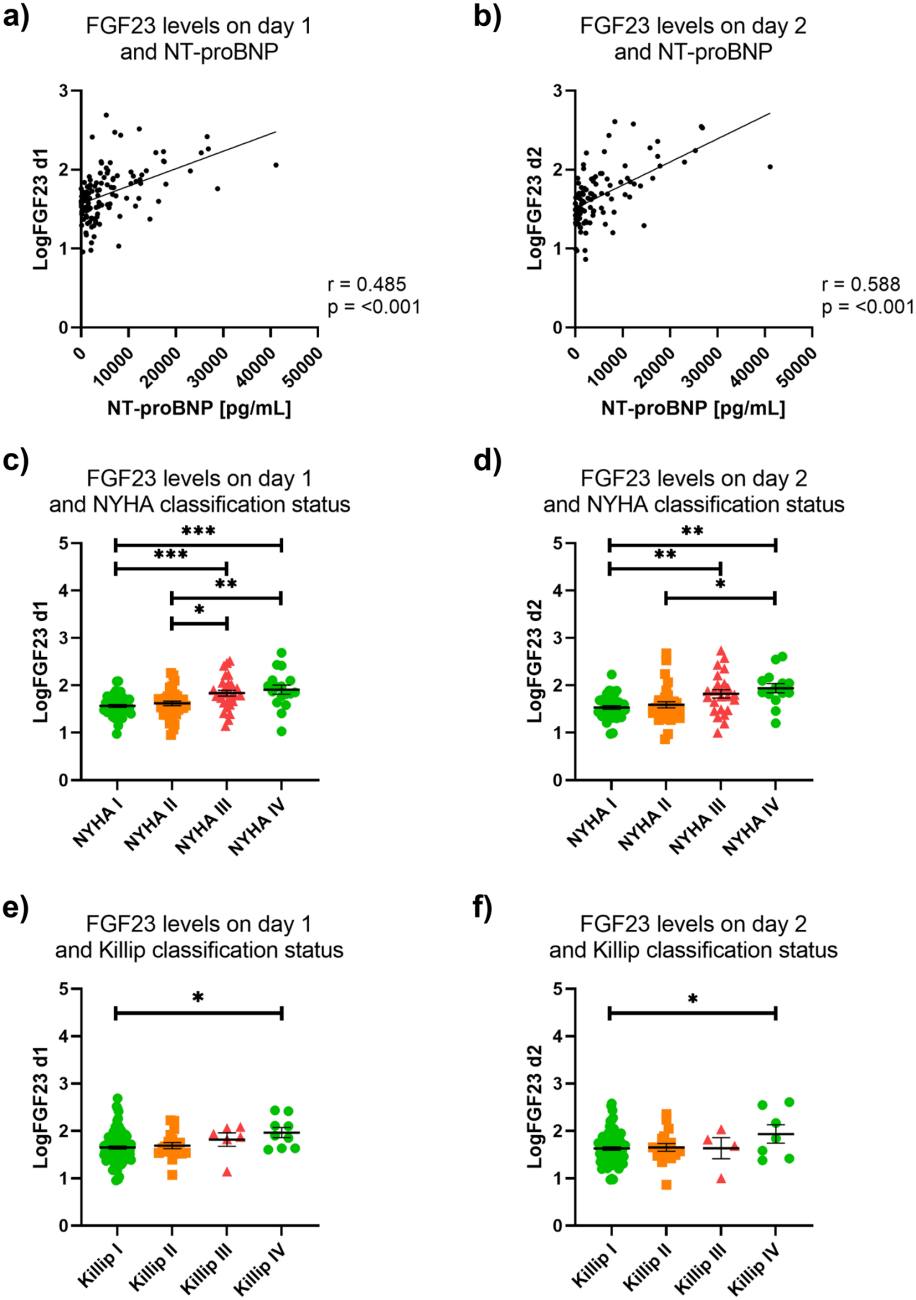
Association between FGF23 levels and severity of heart failure. (**a**, **b**) FGF23 levels were positively correlated with NT-proBNP levels, both when FGF23 was assessed on day 1 (**a**) and on day 2 (**b**). (**c–f**) Indicating associations between FGF23 levels and clinical severity of HF, FGF23 levels increased with worsening NYHA (**c**, **d**) and Killip classification status (**e**, **f**).

Increased NT-proBNP levels can serve as a marker for the clinical severity of the disease^12^. Thus, given the significantly increased logFGF23 levels in patients with preserved LVEF, we investigated whether logFGF23 showed a robust correlation with NT-proBNP rather than LVEF when adjusted for confounding covariates. On day 1 and day 2 after admission for acute HF, logFGF23 levels were positively correlated with NT-proBNP levels, both in an unadjusted analysis (day 1: r = 0.49, *p* =  < 0.001; day 2: r = 0.59, *p* =  < 0.001; Fig. 2a,b) and after controlling for age, eGFR, and LVEF (day 1: r = 0.25; *p* = 0.005; day 2: (r = 0.42; *p* =  < 0.001) (Table 1). Furthermore, logFGF23 levels on day 1 and on day 2 increased with worsening NYHA and Killip classification status (Fig. 2c–f, Table 1), suggesting associations between FGF23 levels and clinical severity of HF. LogFGF23 levels on day 1 also showed a robust correlation with echocardiographic functional parameters of HF, including LVEDD, LVESD, and LA area, both in an unadjusted analysis and when adjusted for age, NT-proBNP, eGFR, and LVEF, while we did not observe correlations between logFGF23 levels and septum thickness, deceleration time (DT), E/A, E/E’, RVSP, and TAPSE (Table 1).

### Elevated FGF23 levels are associated with a higher mortality in patients with acute heart failure

At one-year follow-up, 23 patients had died (17.3%) and 110 (82.7%) out of 133 HF patients were still alive. Patients who died within one year after admission to the Intermediate Care Unit had significantly higher logFGF23 levels than patients who survived this period, both on day 1 and on day 2 in the unadjusted analysis (Supplementary Table S1).

Elevated logFGF23 levels assessed on day 1 still correlated significantly with one-year mortality in a logistic regression analysis, after adjustment for LVEF, eGFR, and NT-proBNP (OR 15.07; 95%CI 1.75–129.79; *p* = 0.014; Table 3). No association with mortality was observed when FGF23 was assessed on day 2 after admission (OR 6.57; 95%CI 0.69–62.69; *p* = 0.102; Table 3). We observed a higher percentage of non-survivors among patients with chronic decompensated HF as compared to patients with de novo HF (Table 1), and patients with chronic decompensated HF were in worse clinical condition as compared to patients with de novo HF (Supplementary Table S2). Importantly, we noticed higher logFGF23 levels in chronic decompensated HF patients. Therefore, we included the condition of having “chronic decompensated HF” as a covariate in the model. Nevertheless, higher logGF23 levels on day 1 were still predictive of mortality (OR 10.33, 95%CI 1.25–85.79; *p* = 0.030; Supplementary Table S3).

**Table 3:** Logistic regression analysis for one-year mortality.

Variables	FGF23 day 1				FGF23 day 2			
	*p* value	Odds ratio Non-survival	95% CI		*p* value	Odds ratio Non-survival	95% CI	
			Lower	Upper			Lower	Upper
LVEF	0.573	0.986	0.938	1.036	0.438	0.978	0.924	1.035
eGFR	0.745	1.003	0.983	1.024	0.783	1.003	0.982	1.025
NT-proBNP	0.027	1.000	1.000	1.000	0.036	1.000	1.000	1.000
logFGF23	0.014	15.073	1.751	129.79	0.102	6.565	0.687	62.689

### FGF23 levels predict one-year mortality as accurately as the seattle heart failure model

**Figure 3:**
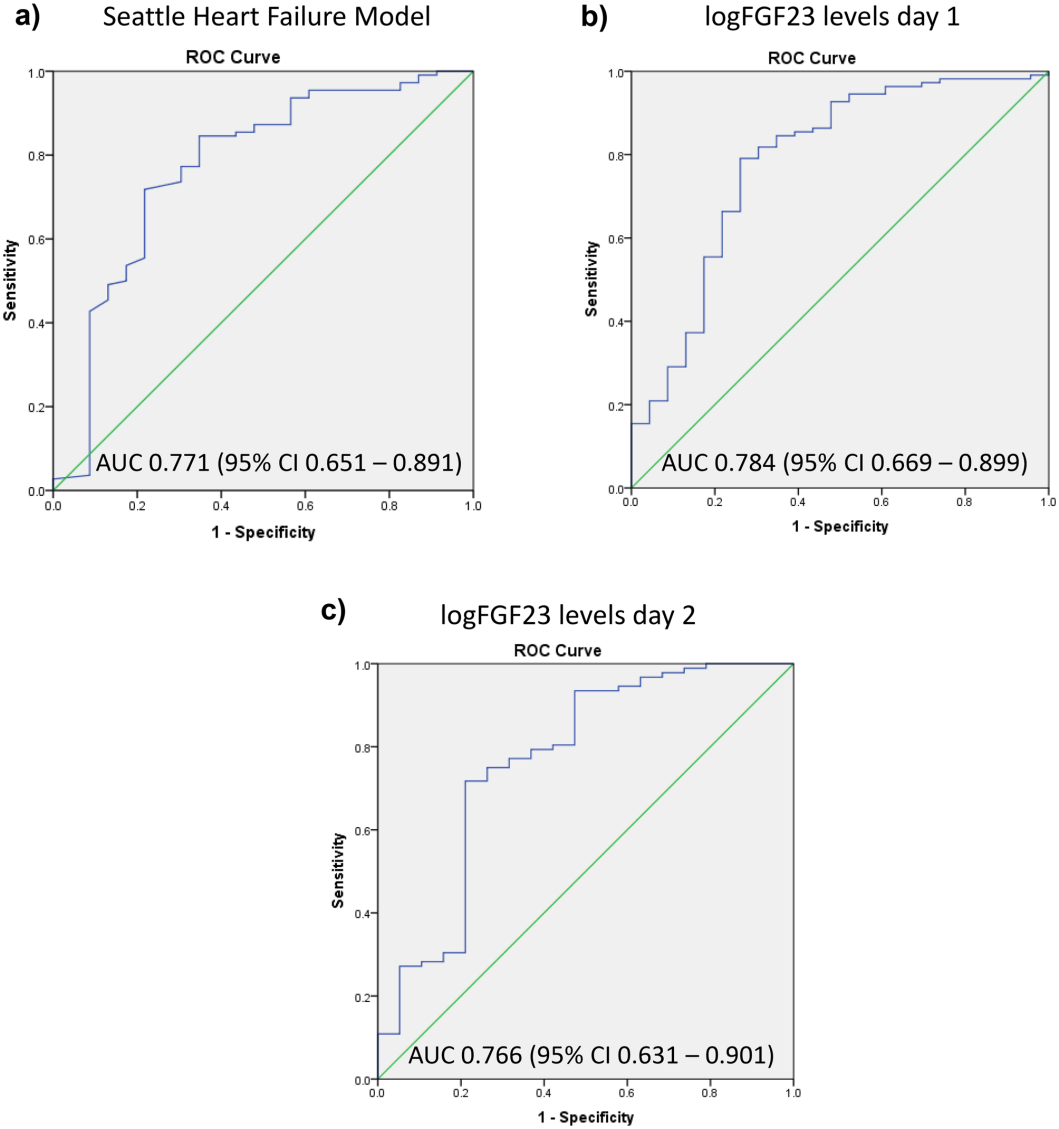
Prediction of the one-year endpoint in patients with acute heart failure. (**a**) The SHF Model provided a fair discrimination between patients who survived and patients who died within the first year after hospital admission. (**b**, **c**) LogFGF23 levels on day 1 (**b**) and on day 2 (**c**) predicted survival with similar accuracy as the SHF Model.

The SHF model correctly classified one-year survival in 82.0% of HF patients with a sensitivity of 95.5% and a specificity of 17.4%. Higher SHF scores were associated with a higher likelihood of survival (OR 1.04; 95%CI 1.02–1.06; *p* =  < 0.0001), and the AUC value of 0.771 (95%CI 0.651–0.891) showed a fair discrimination between patients who survived and patients who died within the first year after hospital admission (Fig. 3a). LogFGF23 levels on day 1 and on day 2 showed similar accuracy for the prediction of the one-year outcome in HF patients (logFGF23 day 1, AUC 0.784; 95%CI 0.669–0.899; sensitivity 97.3%, specificity 26.1%; logFGF23 day 2, AUC 0.766; 95%CI 0.631–0.901; sensitivity 96.7%, specificity 31.6%, Fig. 3b,c). We evaluated by logistic regression whether the addition of logFGF23 to the SHF score increased the percentage accuracy in classification. Addition of day 1-logFGF23 led to slightly increased sensitivity (96.4%) and specificity of the model (30.4%). Overall, the model including SHF scores and logFGF23 on day 1 correctly classified 85.0% of cases. Addition of logFGF23 levels on day 2 to the SHF model further increased specificity (42.1%) and sensitivity (97.8%), resulting in 88.3% accuracy in classification.

## Discussion

In the present study we showed that elevated serum concentrations of FGF23 were associated with one-year mortality in critically ill patients with acute HF admitted to an intermediate care unit of a tertiary referral hospital. The ability of FGF23 levels to discriminate between survivors and non-survivors was similar to the well-established SHF Model. Moreover, addition of FGF23 levels to SHF led to increased accuracy in classification.

Risk stratification is indispensable for the clinical management of HF patients. Several models have been developed to standardize prognosis estimation. Such models usually comprise a set of demographics, laboratory, and imaging data, eventually producing a risk estimation score. However, the assessment of all variables contributing to a prognostic model can be challenging and time-consuming, especially in emergency situations. Therefore, in daily practice, the estimate of a patient’s prognosis in acute situations is often mainly based on the clinician’s experience.

Here, we showed for the first time that the assessment of FGF23 levels offers similar accuracy in prognosis estimation as the SHF model in patients hospitalized for acute HF. Since FGF23 levels can be easily assessed from the patients’ serum, FGF23 might serve as a risk estimation tool that is especially valuable in emergency situations.

While FGF23 is a strong predictor of mortality in dialysis patients^13^, elevated FGF23 levels have also been associated with an increased mortality in non-CKD populations, mainly driven by a higher risk for cardiovascular events^5,9^. It is well-known that FGF23 concentrations are elevated in patients with chronic HF^14,15^. Patients with cardiogenic shock have a tremendous increase in FGF23, which has been associated with poor outcome^16,17^.

While FGF23 has been highly praised for predicting HF-related outcome and prognosis in previous studies^6,18–20^, more recent work has challenged its independent predictive value^10,21^. FGF23 has been shown to be a powerful biomarker for onset and progression of CKD^22,23^, and levels rise even before serum creatinine^24^. Thus, it is unclear whether FGF23 elevation might simply represent an early manifestation of impaired kidney function, even if eGFR is seemingly normal.

It is unclear whether FGF23 excess is solely a biomarker or contributes mechanistically to adverse outcomes as only few studies have investigated causality. Although Faul et al. showed in animal models and in isolated rat cardiomyocytes that treatment with FGF23 may directly cause pathological cardiac remodeling^25^, the administration of FGF23 has also been suggested to increase blood pressure^26^, which itself might contribute to left-ventricular hypertrophy. Data from the EVOLVE trial (Evaluation of Cinacalcet HCl Therapy to Lower Cardiovascular Events) suggest lowering FGF23 levels by Cinacalcet is associated with lower rates of cardiovascular death and major cardiovascular events^27^. On the other hand, data on primary elevations of FGF23, as seen in x-linked hypophosphatemia^28^ and cardiovascular morbidity are conflicting^29,30^, and high circulating FGF23 levels did not alter cardiac function in mice^31^. Furthermore, it is unclear if FGF23 elevation in HF represents an effect of HF itself independent of CKD or is a consequence of subclinical reductions in renal perfusion that stimulate FGF23 production through the same pathways as in early CKD^32^.

Nevertheless, recent studies suggest that FGF23 may be produced and secreted by cardiac tissues themselves especially in response to ischemia, pressure overload, and acute decompensated HF, and that locally produced FGF23 stimulates pro-fibrotic factors that promote pathogenic cardiac remodeling^11,33^. Interestingly, FGF23 levels were higher in patients with HFpEF compared with HFmrEF patients in our study, and FGF23 levels were not associated with LVEF after adjustment for age, NT-proBNP, and eGFR. Instead, FGF23 levels correlated with more functional echocardiographic parameters of HF, such as LVEDD, LVESD, and LA area, even when LVEF was controlled for. Of note, this correlation was observed even though echocardiography was not performed at the worst state of decompensation, but within the first 72 h after admission when some patients in part may have recompensated. FGF23 has recently been shown to be higher in patients with HFpEF compared to subjects with normal LVEF, but without signs or symptoms of HF^34^. In line with previous studies^20,34^, FGF23 also correlated with NYHA and Killip classification status as clinical indicators of HF severity, albeit with the important caveat that most patients in our study had no or mild limitations of physical activity before admission (i.e., 65.4% of patients were NYHA I or II), and the vast majority (88.7% of patients) had no overt pulmonary edema (Killip class III) or signs of cardiogenic shock (Killip class IV) at admission.

However, while high FGF23 levels were consistently associated with a modestly increased risk of cardiovascular death, they were also associated with an increased risk of non-cardiovascular causes of death in a recent meta-analysis, suggesting that the relationship between FGF23 and CVD risk may be non-causal^35^. We did not distinguish between cardiovascular and non-cardiovascular causes of death, which is certainly a limitation in our study. It is also noteworthy that the odds ratio for non-survival for FGF23 was an order of magnitude higher than other metrics, suggesting FGF23 might indeed be a powerful marker of worse outcome in acute heart failure. However, at the same time, the wide 95% confidence interval suggests a great variance and therefore some uncertainty. Importantly, our sample size was rather small, and the single-center design of our study limits the generalizability of our findings. Therefore, larger studies, ideally in a multicentric setting, are necessary to validate FGF23 as a prognostic biomarker in acute HF. In addition, our study cohort was largely male, which, however, might be explained by the higher incidence and prevalence of HF in men that has been reported at all ages^36^.

**Figure 4:**
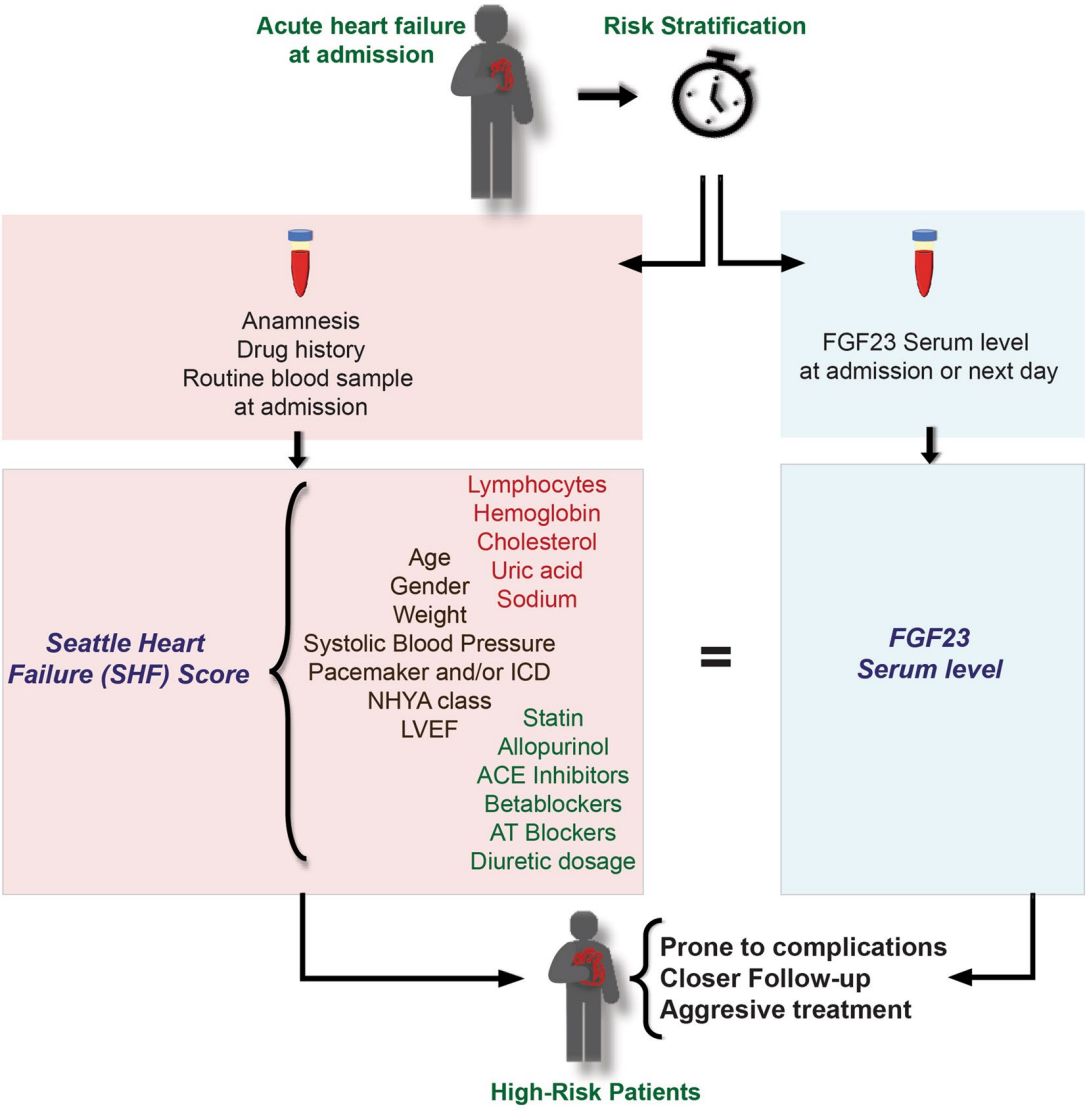
FGF23 levels predict the one-year outcome in patients with acute HF with similar accuracy as the SHF Model. This might be of interest especially in emergency situations, where the assessment of all variables contributing to a prognostic model can be challenging and time-consuming. The figure was created in Adobe Illustrator (Version 25.2.3; https://www.adobe.com/products/illustrator.html).

Although it is possible that elevated FGF23 levels simply reflect the loss of filtration capacity rather than being an independent biomarker in patients with acute HF, we showed that FGF23 predicted one-year survival as accurately as the SHF Model in critically ill HF patients, with even higher accuracy by combining FGF23 and SHF. Because FGF23 is relatively easy to assess, it might be especially valuable in emergency situations (Fig. 4).

## Conclusion

FGF23 levels were similarly accurate as the SHF Model in predicting survival at one year after hospitalization for acute HF in a critically ill patient cohort. Incremental accuracy for mortality prediction can be achieved by combining FGF23 levels with the SHF Model. The assessment of FGF23 levels might help identify patients who are more prone to complications, need a closer follow-up, and a more aggressive treatment.

## Methods

### Study population

A total of 139 patients admitted to the Intermediate Care Unit of University Hospital RWTH Aachen for acute HF between August 2016 and September 2018 were included in the study. Exclusion criteria were age < 18 years, pregnancy or breastfeeding, CKD requiring dialysis, heart or kidney transplantation, and primary admission to the Intensive Care Unit or transfer to the Intensive Care Unit within 12 h after admission to the Intermediate Care Unit. Furthermore, patients who died within 12 h after admission were excluded from the study. In accordance with the working hours of our clinical study center, patients were screened from Mondays–Thursdays between 7 am and 1 pm. The Independent Ethics Committee at the RWTH Aachen Faculty of Medicine approved the study protocol (EK 099/16), and all methods were in accordance with the Declaration of Helsinki and current guidelines. Written informed consent was obtained before study enrollment from all patients.

### Clinical definition of acute heart failure

HF was defined in accordance with the current ESC guidelines^37^. Acute HF included patients with acute de novo HF and decompensated chronic HF. HF with preserved left-ventricular ejection fraction (LVEF) (HFpEF) was defined as LVEF ≥ 50%, HF with midrange LVEF (HFmrEF) was defined as an LVEF 40–49%, and HF with reduced EF (HFrEF) was defined as an LVEF < 40%. We used the NYHA and Killip functional classifications to describe the severity of symptoms.

### Laboratory analysis

Blood samples were collected at the time of admission (“day 1”) and on the following day (“day 2”). Routine laboratory analyses were performed at our core laboratory facility. In addition, serum levels of intact FGF23 were measured using a fully automated chemiluminescent assay (Liaison XL, DiaSorin S.p.A., Saluggia, Italy).

### Echocardiography

Routine echocardiographic examinations were performed at our echocardiography core facility within the first 72 h after hospitalization. The LVEF was calculated in the apical four- and two-chamber views using the modified biplane Simpson’s rule. Diastolic dysfunction was defined as E/e’ ≥ 13 and a mean e’ (septal and lateral wall) < 9 cm/s^37^. Further measurements included left ventricular end-diastolic diameter (LVEDD), left ventricular end-systolic diameter (LVESD), septum thickness, left atrial area (LA area), right-ventricular systolic pressure (RVSP), and tricuspid annular plane systolic excursion (TAPSE).

### Study endpoints and follow-up

The primary endpoint was to identify any association between FGF23 levels and survival at one year in patients with acute HF. Secondary endpoints aimed to examine FGF23 as a biomarker for prognosis estimation in comparison to the SHF Model. Furthermore, we investigated associations between FGF23 and clinical and echocardiographic parameters of HF severity. All patients were followed up by phone calls to assess their survival at one year after admission.

### Risk calculation

We used the SHF Model^38^ to calculate the anticipated 1-year survival (https://qxmd.com/calculate/calculator_203/seattle-heart-failure-model). Variables included in the model were age, LVEF, systolic blood pressure, weight, sex, NYHA class, etiology (ischemic vs. non-ischemic), dosages of furosemide, torsemide, bumetidine, metolazone, and HCTZ, usage of allopurinol, statins, ACE inhibitors, beta-blockers, angiotensin-receptor blockers, K-sparing diuretics, the presence of biventricular pacemakers, implantable cardioverter defibrillators, or combined devices, and blood levels of sodium, cholesterol, hemoglobin, lymphocytes, and uric acid.

### Statistical analysis

Baseline characteristics were assessed by standard descriptive statistics. Continuous data are expressed as mean ± SD. Categorical data are presented as numbers and percentages. Shapiro–Wilk test was used to assess normality. Serum FGF23 levels were not normally distributed and therefore log transformed (logFGF23). Potential outliers were identified using the ROUT method, as described elsewhere^39^, based on a false discovery rate of 1%. Statistical differences between continuous variables were determined with student’s t-test or one-way ANOVA, followed by Tukey’s Multiple Comparisons testing, and between categorical variables with Chi Square Test. We used Pearson’s correlation to investigate the relationship between logFGF23 and LVEF, and between logFGF23 and NT-proBNP, and partial correlation was used to control for age, eGFR, NT-proBNP and/or LVEF. Direct logistic regression analyses were performed to assess associations between logFGF23 and survival at one year. Each model was adjusted for LVEF, eGFR, and NT-proBNP. Furthermore, we calculated areas under the ROC curve (AUCs) and the corresponding 95%-confidence intervals (CI) to compare the performances of the SHF Model and FGF23 for mortality prediction. All statistical analyses were performed using IBM SPSS statistics, version 22.0. Graphs were created in GraphPad Prism (GraphPad Software, Version 8.4.1). Figure 4 was created in Adobe Illustrator (Version 25.2.3).

## Supplementary Information

Supplementary Information.

Supplementary Figure.

## Data Availability

All data is available from the corresponding author upon reasonable request.

## Abbreviations

CVD: Cardiovascular diseases
FGF23: Fibroblast growth factor 23
CKD: Chronic kidney disease
HF: Heart failure
ACE inhibitor: Angiotensin-converting enzyme inhibitor
SHF: Seattle Heart Failure Model
LVEF: Left-ventricular ejection fraction
HFpEF: Heart failure with preserved ejection fraction
HFmrEF: Heart failure with midrange ejection fraction
HFrEF: Heart failure with reduced ejection fraction
LVEDD: Left ventricular end-diastolic diameter
LVESD: Left ventricular end-systolic diameter
LA area: Left atrial area
RVSP: Right-ventricular systolic pressure
TAPSE: Tricuspid annular plane systolic excursion
DT: Deceleration time
AUC: Area under the ROC curve
