# Supplementary material for: Intact fibroblast growth factor 23 levels and outcome prediction in patients with acute heart failure
Source: Sci Rep. 2021 Jul 30;11:15507. doi: 10.1038/s41598-021-94780-7 (PMC8324826; doi:10.1038/s41598-021-94780-7)
Supplement: Supplementary file 1 — Supplementary Information. [file 41598_2021_94780_MOESM1_ESM.docx]

Intact fibroblast growth factor 23 levels and outcome prediction in patients with acute heart failure

Anne Cornelissen^1^, Roberta Florescu^1^, Kinan Kneizeh^1^, Christian Cornelissen^2^, Vincent Brandenburg^3^, Elisa Liehn^1^, Alexander Schuh^4^*

**Brief title:** FGF23 predicts outcome in acute heart failure

^1^ Department of Cardiology, Angiology and Internal Intensive Medicine, University Hospital Aachen, RWTH Aachen University, Germany

^2^ Department of Pneumology, University Hospital Aachen, RWTH Aachen University, Germany

^3^ Department of Cardiology and Nephrology, Rhein-Maas Klinikum, Wuerselen, Germany

^4^ Department of Internal Medicine I, St. Katharinen Hospital Frechen, Germany

***Address for correspondence:**

Alexander Schuh, MD

St. Katharinen Hospital Frechen

Kapellenstrasse 1-5

50226 Frechen

Germany

Phone: +49 2234 502-28110

FAX: +49 2234 502-28104

aschuh@ukaachen.de

**Supplementary Figure S1: Study Cohort.** From a total of 139 patients with acute HF, FGF23 levels were available in 137 patients. The final study cohort encompassed a total of 133 patients with completed one-year follow-up, including 96 patients with *de novo* HF and 37 patients presenting with chronic decompensated HF. At one-year follow-up, 23 patients had died, and 110 patients were still alive.

**Supplementary Table S1:** Patient Characteristics in Survivors vs. Non-Survivors with Acute HF at 1 Year

|  | **Overall Cohort**  **(n=133)** | **Survival 1 year**  **(n = 110)** | **Death 1 year**  **(n = 23)** | **p-value** |
| --- | --- | --- | --- | --- |
| Age, years (Range) | 66.98 (32 – 94) | 66.23 (35 – 94) | 70.57 (32 – 90) | 0.122 |
| Male sex, n (%) | 96 (72.2%) | 77 (70.0%) | 19 (82.6%) | 0.220 |
| BMI, kg/m^2^ | 27.38 ± 5.10 | 27.28 ± 4.91 | 27.85 ± 6.03 | 0.622 |
| Systolic Blood Pressure, mmHg | 119.90 ± 24.30 | 120.94 ± 24.39 | 114.96 ± 23.80 | 0.285 |
| Heart Rate, bpm | 82.81 ± 19.47 | 83.38 ± 19.90 | 80.09 ± 17.38 | 0.462 |
|  | | | | |
| *De novo* HF, n (%) | 96 (72.2%) | 86 (78.2%) | 10 (43.5%) | 0.001 |
| Chronic Decompensated HF, n (%) | 37 (27.8%) | 24 (21.8%) | 13 (56.5%) | 0.001 |
| LVEF | | | | |
| ≥50%, n (%) | 9 (6.8%) | 7 (6.4%) | 2 (8.7%) | 0.686 |
| 40 – 49%, n (%) | 58 (43.6%) | 53 (48.2%) | 5 (21.7%) | 0.020 |
| ≤39%, n (%) | 66 (49.6%) | 50 (45.5%) | 16 (69.6%) | 0.035 |
| LVEF mean ± SD (%) | 38.39 ± 10.30 | 39.51 ± 9.55 | 33.04 ± 12.17 | 0.006 |
| Killip Classification | | | | |
| 1, n (%) | 99 (74.4%) | 86 (78.2%) | 13 (52.0%) | 0.030 |
| 2, n (%) | 19 (14.3%) | 14 (12.7%) | 5 (21.7%) | 0.261 |
| 3, n (%) | 6 (4.5%) | 4 (3.6%) | 2 (8.7%) | 0.288 |
| 4, n (%) | 9 (6.8%) | 6 (5.5%) | 3 (13.0%) | 0.188 |
| NYHA Classification | | | | |
| 1, n (%) | 50 (37.6%) | 47 (42.7%) | 3 (13.0%) | 0.008 |
| 2, n (%) | 37 (27.8%) | 30 (27.3%) | 7 (30.4%) | 0.758 |
| 3, n (%) | 29 (21.8%) | 22 (20.0%) | 7 (30.4%) | 0.270 |
| 4, n (%) | 17 (12.8%) | 11 (10.0%) | 6 (26.1%) | 0.036 |
| Echocardiography | | | | |
| LVEDD (mm) | 52.42 ± 8.21 | 51.56 ± 7.95 | 57.67 ± 8.05 | 0.005 |
| LVESD (mm) | 40.93 ± 10.35 | 39.88 ± 9.93 | 46.90 ± 11.14 | 0.012 |
| Septum (mm) | 10.87 ± 1.90 | 10.90 ± 1.93 | 10.69 ± 1.82 | 0.681 |
| LA area (mm^2^) | 21.69 ± 5.94 | 20.82 ± 5.46 | 26.68 ± 6.08 | <0.001 |
| DT (ms) | 197.70 ± 56.65 | 198.94 ± 57.20 | 183.24 ± 50.37 | 0.459 |
| E/A | 1.14 ± 0.55 | 1.15 ± 0.57 | 1.10 ± 0.27 | 0.859 |
| E/E’ | 13.09 ± 5.25 | 12.87 ± 4.89 | 15.67 ± 8.66 | 0.212 |
| RVSP (mmHg) | 32.42 ± 12.86 | 31.32 ± 11.15 | 38.64 ± 19.54 | 0.082 |
| TAPSE (mm) | 2.12 ± 1.29 | 2.18 ± 1.36 | 1.78 ± 0.55 | 0.188 |
| Diastolic Dysfunction | | | | |
| 0, n (%) | 12 (9.0%) | 11 (10.0%) | 1 (4.3%) | 0.390 |
| 1, n (%) | 28 (21.1%) | 27 (24.5%) | 1 (4.3%) | 0.031 |
| 2, n (%) | 28 (21.1%) | 25 (22.7%) | 3 (13.0%) | 0.300 |
| 3, n (%) | 8 (6.0%) | 8 (7.3%) | 0 (0.0%) | NA |
| Unknown, n (%) | 57 (42.9%) | 39 (35.5%) | 18 (78.3%) | <0.001 |
| Medication | | | | |
| Beta blockers, n (%) | 122 (91.7%) | 105 (95.5%) | 17 (73.9%) | 0.001 |
| ACE inhibitors / Angiotensin II Receptor Blockers, n (%) | 124 (93.2%) | 104 (94.5%) | 20 (87.0%) | 0.188 |
| Statins, n (%) | 122 (91.7%) | 103 (93.6%) | 19 (82.6%) | 0.081 |
| Loop Diuretics, n (%) | 80 (60.2%) | 60 (54.5%) | 20 (87.0%) | 0.004 |
| Risk Factors | | | | |
| Hypertension, n (%) | 77 (57.9%) | 61 (55.5%) | 16 (69.6%) | 0.213 |
| Smoking, n (%) | 76 (56.3%) | 63 (57.3%) | 13 (52.0%) | 0.947 |
| Diabetes, n (%) | 41 (30.8%) | 27 (24.5%) | 14 (60.9%) | 0.001 |
| Hypercholesterolemia, n (%) | 76 (57.1%) | 60 (54.5%) | 16 (69.6%) | 0.186 |
| Chronic kidney disease, n (%) | | | | |
| CKD Level 1, n (%) | 50 (37.6%) | 46 (41.8%) | 4 (17.4%) | 0.028 |
| CKD Level 2, n (%) | 39 (29.3%) | 35 (31.8%) | 4 (17.4%) | 0.167 |
| CKD Level 3, n (%) | 34 (25.6%) | 24 (21.8%) | 10 (43.5%) | 0.030 |
| CKD Level 4, n (%) | 9 (6.8%) | 4 (3.6%) | 5 (21.7%) | 0.002 |
| CKD Level 5, n (%) | 1 (0.7%) | 1 (0.9%) | 0 (0.0%) | NA |
| Blood Parameters | | | | |
| Total Cholesterol, mg/dL | 168.31 ± 52.43 | 172.14 ± 50.19 | 150.00 ± 59.91 | 0.065 |
| LDL Cholesterol, mg/dL | 111.07 ± 49.98 | 114.78 ± 49.00 | 92.50 ± 51.79 | 0.056 |
| Creatinine, mg/dL | 1.19 ± 0.68 | 1.07 ± 0.59 | 1.75 ± 0.80 | <0.001 |
| Urea, mg/dL | 52.79 ± 40.37 | 44.38 ± 29.03 | 93.00 ± 59.65 | <0.001 |
| Estimated GFR, mL/min/1.73m^2^ | 81.64 ± 38.57 | 86.52 ± 37.08 | 58.28 ± 37.72 | <0.001 |
| Calcium, mmol/L | 2.17 ± 0.15 | 2.19 ± 0.12 | 2.08 ± 0.24 | 0.002 |
| Phosphorus, mmol/L | 1.08 ± 0.29 | 1.04 ± 0.25 | 1.29 ± 0.38 | <0.001 |
| Uric acid, mg/dL | 6.74 ± 2.54 | 6.25 ± 2.08 | 9.12 ± 3.18 | <0.001 |
| Alkaline Phosphatase, U/L | 90.05 ± 72.60 | 84.44 ± 71.10 | 115.91 ± 75.42 | 0.059 |
| CK, U/L | 1,323.66 ± 2,336.04 | 1,456.48 ± 2,482.34 | 694.22 ± 1,320.22 | 0.156 |
| CK-MB, U/L | 135.94 ± 167.40 | 148.66 ± 174.86 | 74.07 ± 107.89 | 0.057 |
| Troponin T, pg/mL | 2,548.00 ± 3,750.64 | 2,743.74 ± 3,804.16 | 1,620.35 ± 3,410.58 | 0.193 |
| NT-proBNP, pg/mL | 5,623.04 ± 6,976.99 | 4,202.27 ± 4,954.99 | 10,629.40 ± 2,319.53 | <0.001 |
| Leukocytes, /nL | 11.62 ± 4.28 | 11.71 ± 4.07 | 11.21 ± 5.28 | 0.617 |
| Hemoglobin, g/dL | 12.80 ± 2.44 | 13.17 ± 2.29 | 11.07 ± 2.43 | <0.001 |
| LogFGF23 day 1 | 1.69 ± 0.32 | 1.63 ± 0.28 | 1.97 ± 0.34 | <0.001 |
| LogFGF23 day 2 | 1.65 ± 0.35 | 1.58 ± 0.29 | 1.99 ± 0.42 | <0.001 |
| Seattle Heart Failure Model | | | | |
| Anticipated 1-year Survival (%) | 85.50 ± 22.86 | 89.69 ± 17.69 | 65.47 ± 32.85 | <0.001 |

**Supplementary Table S2:** Characteristics of Patients with *de novo* HF vs. chronic decompensated HF

|  | ***De novo* HF**  **(n = 96)** | **Chronic Decompensated HF**  **(n = 37)** | **p-value** |
| --- | --- | --- | --- |
| Age, years (Range) | 65.78 ± 11.53 | 70.08 ± 13.54 | 0.069 |
| Male sex, n (%) | 72 (75.0%) | 24 (64.9%) | 0.242 |
| BMI, kg/m^2^ | 27.46 ± 4.81 | 27.15 ± 5.86 | 0.752 |
| Systolic Blood Pressure, mmHg | 123.69 ± 20.91 | 110.08 ± 29.57 | 0.003 |
| Heart Rate, bpm | 83.90 ± 20.48 | 80.00 ± 16.48 | 0.303 |
|  | | | |
| Survival, n (%) | 86 (89.6%) | 24 (64.9%) | 0.001 |
| Non-Survival, n (%) | 10 (10.4%) | 13 (35.1%) | 0.001 |
| LVEF | | | |
| ≥50%, n (%) | 8 (8.3%) | 1 (2.7%) | 0.247 |
| 40 – 49%, n (%) | 53 (55.2%) | 5 (13.5%) | <0.001 |
| ≤39%, n (%) | 35 (36.5%) | 31 (83.8%) | <0.001 |
| LVEF mean ± SD (%) | 41.41 ± 8.78 | 30.57 ± 9.92 | <0.001 |
| Killip Classification | | | |
| 1, n (%) | 73 (76.0%) | 26 (70.3%) | 0.494 |
| 2, n (%) | 15 (15.6%) | 4 (10.8%) | 0.477 |
| 3, n (%) | 4 (4.2%) | 2 (5.4%) | 0.758 |
| 4, n (%) | 4 (4.2%) | 5 (13.5%) | 0.054 |
| NYHA Classification | | | |
| 1, n (%) | 48 (50.0%) | 2 (5.4%) | <0.001 |
| 2, n (%) | 28 (29.2%) | 9 (24.3%) | 0.577 |
| 3, n (%) | 14 (14.6%) | 15 (40.5%) | 0.001 |
| 4, n (%) | 6 (6.3%) | 11 (29.7%) | <0.001 |
| Echocardiography | | | |
| LVEDD (mm) | 50.04 ± 7.13 | 60.08 ± 6.72 | <0.001 |
| LVESD (mm) | 37.45 ± 8.08 | 51.50 ± 9.48 | <0.001 |
| Septum (mm) | 11.01 ± 1.87 | 10.44 ± 1.99 | 0.181 |
| LA area (mm^2^) | 20.16 ± 5.33 | 26.46 ± 5.24 | <0.001 |
| DT (ms) | 202.81 ± 56.96 | 157.33 ± 34.30 | 0.022 |
| E/A | 1.15 ± 0.56 | 1.11 ± 0.48 | 0.887 |
| E/E’ | 12.18 ± 4.10 | 18.55 ± 7.84 | <0.001 |
| RVSP (mmHg) | 31.02 ± 10.75 | 35.90 ± 16.80 | 0.143 |
| TAPSE (mm) | 2.05 ± 0.46 | 2.29 ± 2.49 | 0.412 |
| Diastolic Dysfunction | | | |
| 0, n (%) | 12 (12.5%) | 0 (0.0%) | 0.024 |
| 1, n (%) | 26 (27.1%) | 2 (5.4%) | 0.006 |
| 2, n (%) | 24 (25.0%) | 4 (10.8%) | 0.072 |
| 3, n (%) | 7 (7.3%) | 1 (2.7%) | 0.319 |
| Unknown, n (%) | 27 (28.1%) | 30 (81.1%) | <0.001 |
| Medication | | | |
| Beta blockers, n (%) | 89 (92.7%) | 33 (89.2%) | 0.509 |
| ACE inhibitors / Angiotensin II Receptor Blockers, n (%) | 89 (92.7%) | 35 (94.6%) | 0.698 |
| Statins, n (%) | 91 (94.8%) | 31 (83.8%) | 0.039 |
| Loop Diuretics, n (%) | 44 (45.8%) | 36 (97.3%) | <0.001 |
| Risk Factors | | | |
| Hypertension, n (%) | 54 (56.3%) | 23 (62.2%) | 0.536 |
| Smoking, n (%) | 53 (62.2%) | 23 (62.2%) | 0.468 |
| Diabetes, n (%) | 24 (25.0%) | 17 (45.9%) | 0.019 |
| Hypercholesterolemia, n (%) | 54 (56.3%) | 22 (59.5%) | 0.738 |
| Chronic kidney disease, n (%) | | | |
| CKD Level 1, n (%) | 45 (46.9%) | 5 (13.5%) | <0.001 |
| CKD Level 2, n (%) | 30 (31.3%) | 9 (24.3%) | 0.432 |
| CKD Level 3, n (%) | 17 (17.7%) | 17 (45.9%) | 0.001 |
| CKD Level 4, n (%) | 4 (4.2%) | 5 (13.5%) | 0.054 |
| CKD Level 5, n (%) | 0 (0.0%) | 1 (2.7%) | NA |
| Blood Parameters | | | |
| Total Cholesterol, mg/dL | 180.38 ± 46.50 | 137.00 ± 54.58 | <0.001 |
| LDL Cholesterol, mg/dL | 122.92 ± 46.41 | 79.46 ± 45.71 | <0.001 |
| Creatinine, mg/dL | 1.05 ± 0.48 | 1.56 ± 0.93 | <0.001 |
| Urea, mg/dL | 42.43 ± 30.45 | 79.68 ± 49.98 | <0.001 |
| Estimated GFR, mL/min/1.73m^2^ | 90.14 ± 38.10 | 59.57 ± 30.48 | <0.001 |
| Calcium, mmol/L | 2.17 ± 0.16 | 2.17 ± 0.13 | 0.895 |
| Phosphorus, mmol/L | 1.04 ± 0.29 | 1.19 ± 0.25 | 0.014 |
| Uric acid, mg/dL | 6.11 ± 2.23 | 8.39 ± 2.57 | <0.001 |
| Alkaline Phosphatase, U/L | 76.13 ± 35.09 | 127.46 ± 120.41 | <0.001 |
| CK, U/L | 1,659.95 ± 2,591.03 | 426.89 ± 1,032.21 | 0.006 |
| CK-MB, U/L | 163.08 ± 174.61 | 60.10 ± 117.49 | 0.002 |
| Troponin T, pg/mL | 3,077.22 ± 3,813.55 | 1,136.75 ± 3,218.81 | 0.008 |
| NT-proBNP, pg/mL | 4,651.94 ± 6,988.24 | 7,958.92 ± 6,458.10 | 0.015 |
| Leukocytes, /nL | 11.70 ± 3.96 | 11.43 ± 5.08 | 0.746 |
| Hemoglobin, g/dL | 13.33 ± 2.33 | 11.45 ± 2.23 | <0.001 |
| LogFGF23 day 1 | 1.61 ± 0.28 | 1.87 ± 0.34 | <0.001 |
| LogFGF23 day 2 | 1.56 ± 0.29 | 1.88 ± 0.38 | <0.001 |
| Seattle Heart Failure Model | | | |
| Anticipated 1-year Survival (%) | 89.67 ± 20.33 | 74.67 ± 25.67 | 0.001 |

**Supplementary Table S3:** Logistic Regression Analysis of One-Year Mortality with Additional Adjustment for Chronic Decompensated HF

| Variables | FGF23 day 1 | | | | FGF23 day 2 | | | |
| --- | --- | --- | --- | --- | --- | --- | --- | --- |
|  | p-value | Odds Ratio  Non-Survival | 95% C.I. | | p-value | Odds Ratio  Non-Survival | 95% C.I. | |
|  |  |  | Lower | Upper |  |  | Lower | Upper |
| LVEF | 0.547 | 1.018 | 0.961 | 1.078 | 0.958 | 1.002 | 0.939 | 1.068 |
| eGFR | 0.441 | 1.008 | 0.988 | 1.028 | 0.550 | 1.006 | 0.985 | 1.028 |
| NT-proBNP | 0.006 | 1.000 | 1.000 | 1.000 | 0.014 | 1.000 | 1.000 | 1.000 |
| Chronic Decompensated HF | 0.026 | 4.795 | 1.201 | 19.139 | 0.120 | 3.328 | 0.732 | 15.128 |
| logFGF23 | 0.030 | 10.325 | 1.249 | 85.794 | 0.176 | 4.632 | 0.503 | 42.665 |
