# Supplementary material for: Intact fibroblast growth factor 23 levels and outcome prediction in patients with acute heart failure
Source: Sci Rep. 2021 Jul 30;11:15507. doi: 10.1038/s41598-021-94780-7 (PMC8324826; doi:10.1038/s41598-021-94780-7)
Supplement: Supplementary file 2 — Supplementary Figure. [file 41598_2021_94780_MOESM2_ESM.pptx]

## Slide 1
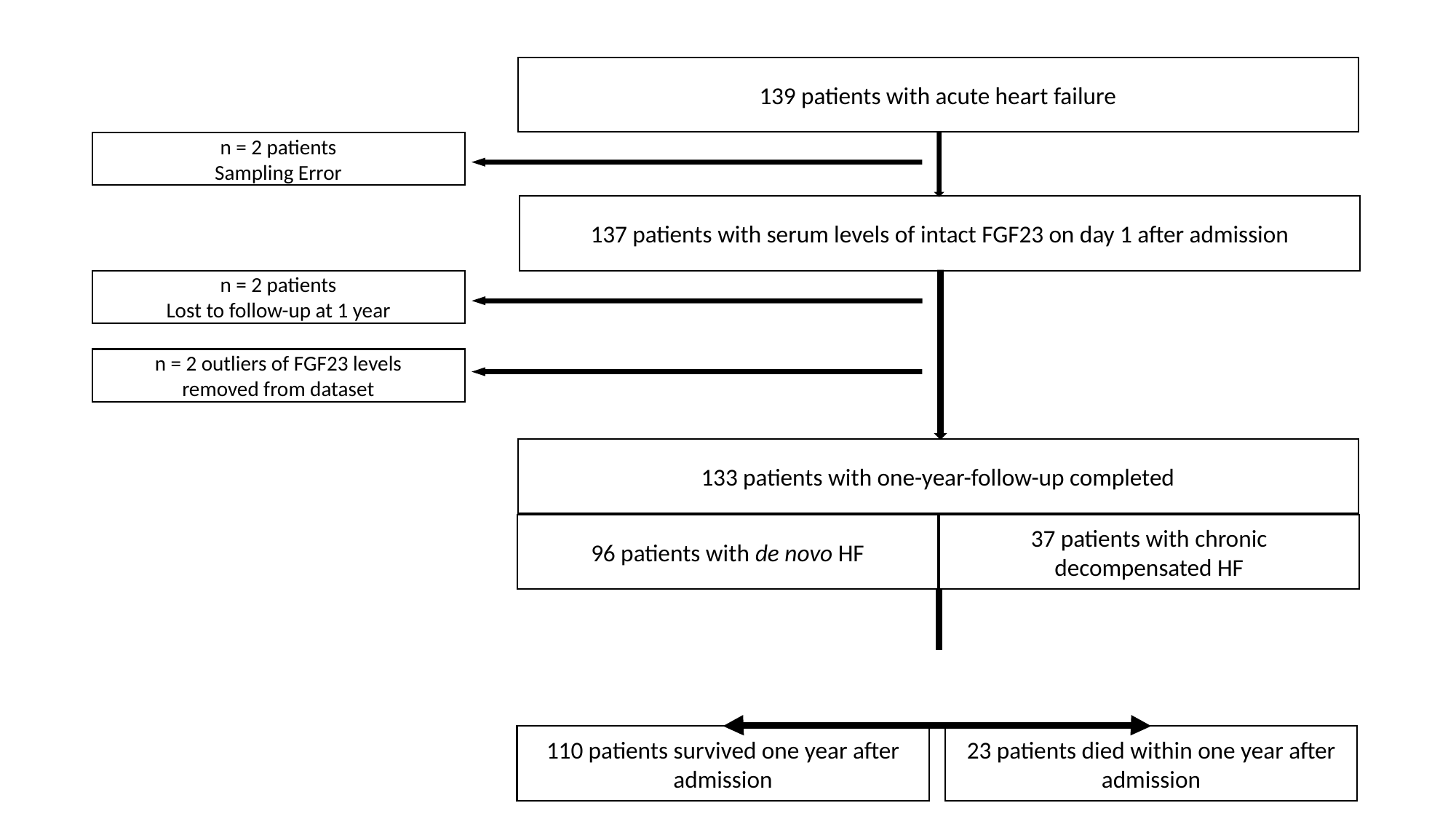

139 patients with acute heart failure
n = 2 patients
Sampling Error
137 patients with serum levels of intact FGF23 on day 1 after admission
n = 2 patients
Lost to follow-up at 1 year
n = 2 outliers of FGF23 levels
removed from dataset
133 patients with one-year-follow-up completed
96 patients with de novo HF
37 patients with chronic decompensated HF
110 patients survived one year after admission
23 patients died within one year after admission
